# Angiomatoid Fibrous Histiocytoma: A Series of Three Cases

**DOI:** 10.7759/cureus.16465

**Published:** 2021-07-18

**Authors:** Mohammed A Alzahim, Abdulaziz H Abed, Hosam T Mashrah, Akeel M Almahdaly, Mahmood Shaheen

**Affiliations:** 1 College of Medicine, King Saud University, Riyadh, SAU; 2 Medicine and Surgery, Alfaisal University College of Medicine, Riyadh, SAU; 3 Medicine and Surgery, Taif University, Taif, SAU; 4 Orthopaedics, King Saud Medical City, Riyadh, SAU; 5 Orthopaedics, King Faisal Specialist Hospital and Research Centre, Riyadh, SAU

**Keywords:** angiomatoid, fibrous, histiocytoma, angiomatoid fibrous histiocytoma, case series, case report

## Abstract

Angiomatoid fibrous histiocytoma (AFH) is a rare, slow-growing soft tissue tumor with an intermediate biologic potential and uncertain line of differentiation, and minimal metastatic potential. AFH may mimic both the clinical, histological, and radiological findings of several tumors; therefore, it is frequently misdiagnosed.

Three cases of AFH were included in this study. A six-year-old male and two females with an age of 12 and 17 years are presented. The primary locations were in the right shoulder (case 1), left medial proximal thigh (case 2), and left lateral knee (case 3). Two cases (cases 2&3) were presented with a painful mass. In the three patients, the masses were firm, mobile, and not attached to the skin.

Magnetic resonance imaging (MRI) was done, illustrating unspecific findings to reach a diagnosis. Needle biopsies were performed in all patient, and the diagnosis of AFH was reached. All three patients underwent wide surgical excision of the tumor. Patients were followed up routinely every three to four months with imaging studies to rule out recurrence and metastasis, for a period of 15 months (case 1), 26 months (case 2), and 19 months (case 3), which all resulted negatively.

## Introduction

Angiomatoid fibrous histiocytoma (AFH) is a rare, slow-growing soft tissue tumor with an intermediate biologic potential and uncertain line of differentiation, and minimal metastatic potential [1,2,3]. AFH may mimic both the clinical, histological, and radiological findings of several tumors; therefore, it is frequently misdiagnosed [3]. The main presentation is painless soft tissue mass, accompanied by systemic symptoms, like anemia, weight loss, and fever, although atypical presentations have been reported [4].

AFH can also present as a slowly growing, superficial nodular mass that is rarely causing pain or tenderness, a matter that makes it misdiagnosed as a hematoma or a hemangioma [5]. It could also be presented by symptoms related to the anatomic site [6]. Molecular studies play a valuable role in confirming the diagnosis of AFH in adjunct to clinical and pathological findings, where the rearrangement of Ewing sarcoma breakpoint region one gene (EWSR1) being a positive finding in (83%) of the cases [4]. Furthermore, AFH is commonly occurring in the extremities of patients in their first three decades of life, affecting 1 in 100,000 of the population, with slight male predominance [1,7 ]. The variable patterns of AFH could lead to diagnostic difficulties and missing diagnoses if the molecular investigation was not done [8]. Many cases have been reported in the literature; nevertheless, none of them in Saudi Arabia, to the best of our knowledge. We have compiled a series of three cases of AFH at our institution, with two presenting with a painful mass. Herein we are discussing the clinical feature, radiological, histopathological, and cytogenic findings.

## Case presentation

The presented three cases were a six-year-old male (case 1) and two female patients with an age of 12 (case 2) and 17 (case 3) years old. The primary locations were in the shoulder (case 1), medial proximal thigh (case 2), and lateral knee (case 3). Two cases (cases 2&3) were presented with a painful mass. In the three patients, the masses were firm, mobile, not attached to the skin, and no skin discolorations except bluish discoloration in one patient (case 3). The approximate sizes of the tumors were 3x3 cm (case 1) and 4x5 cm (case 2&3). All patients had a full range of motion and no other relevant symptoms.

**Figure 1:**
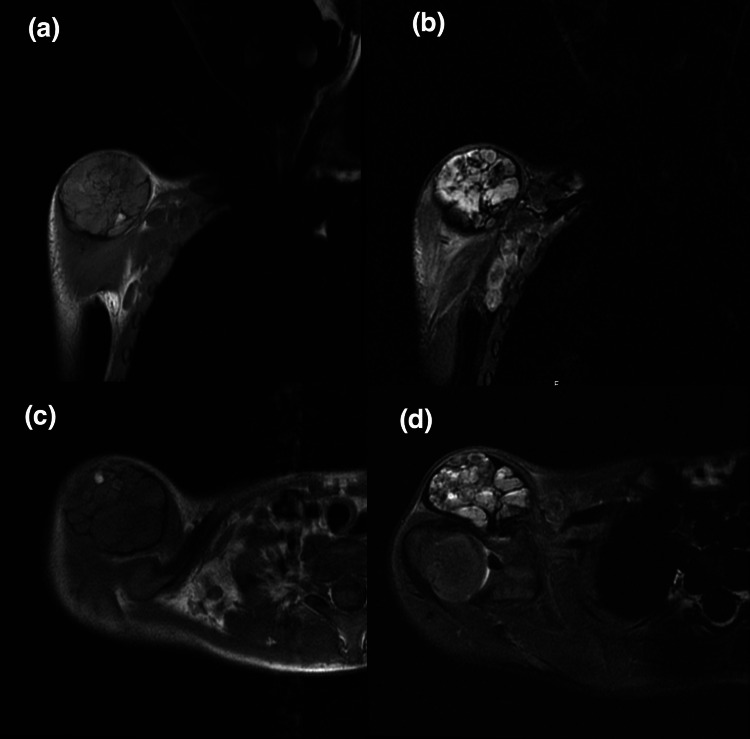
A 6-year-old boy diagnosed with angiomatoid fibrous histiocytoma (case 1): (a) coronal and (c) axial T1-weighted non-fat saturated images. (b) coronal and (d) axial STIR images. A 3.3×2.9×3.1 cm showing multi-speptate cystic lesion in the shoulder. STIR: short T1 Inversion Recovery.

**Figure 2:**
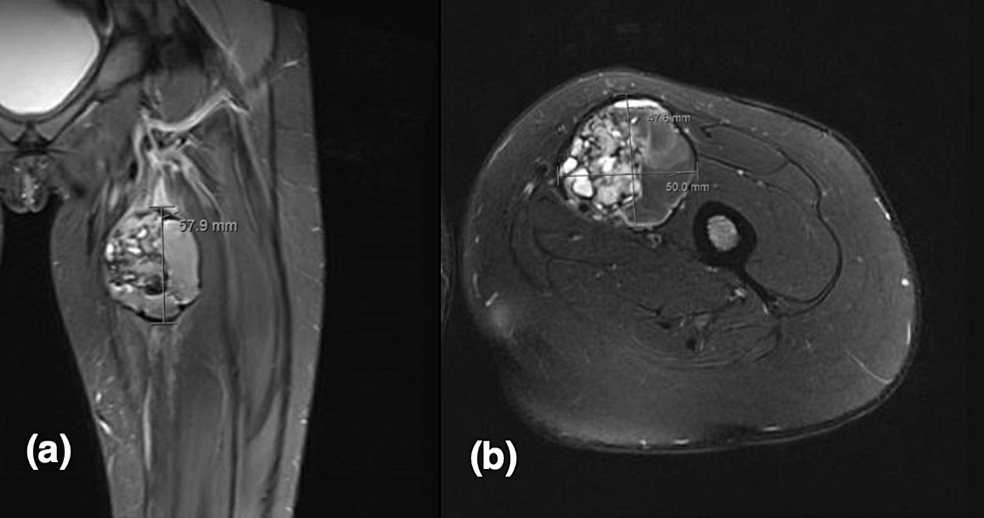
A 12-year-old boy diagnosed with angiomatoid fibrous histiocytoma (case 2): (a) Coronal and (b) axial T2-weighted fat-saturated images. A 5.8x4.8x5.0 cm oval mass showing in the proximal left thigh.

**Figure 3:**
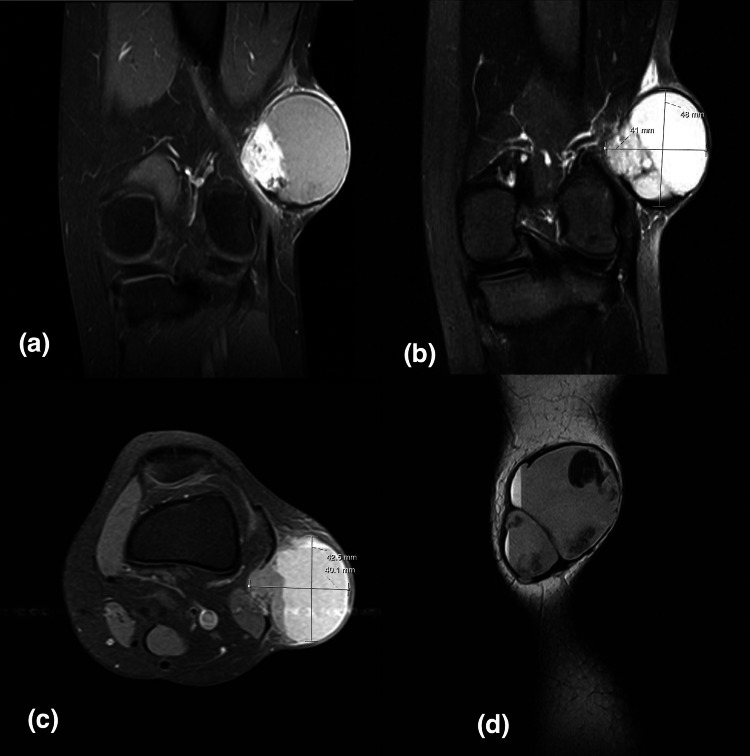
A 17-year-old girl diagnosed with angiomatoid fibrous histiocytoma (case 3): (a) Coronal, (c) axial and sagittal T1-Weighted fat-saturated post-contrast, and (b) Coronal fluid sensitive sequence. A 4.8x4.3x4.0 cm mass showing in the lateral aspect of the left knee.

**Figure 4:**
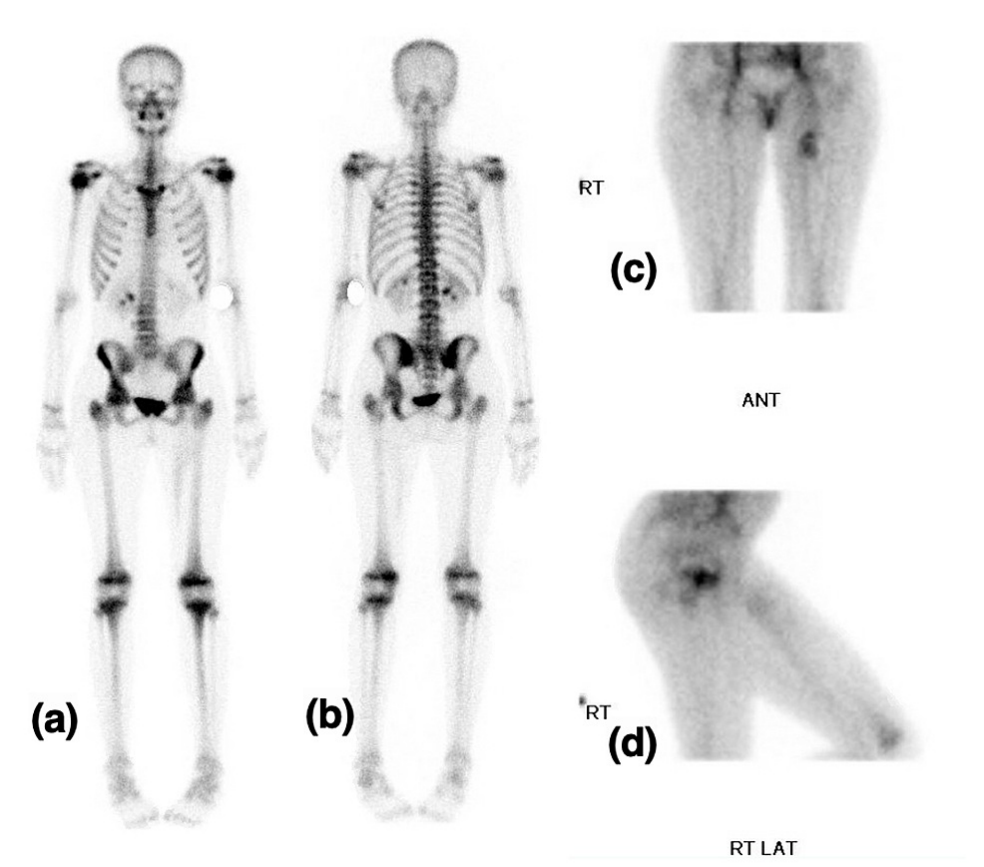
A 12-year-old girl diagnosed with angiomatoid fibrous histiocytoma (case 2): Triphasic Technetium 99 MDP bone scan in (a) anterior view and (b) posterior view of delayed phase. (c) AP view and (c) righ lateral view of blood pooling phase. Mass showing hyperemia in blood pooling phase MDP: methyl diphosphonate; AP: anterior-posterior.

Magnetic resonance imaging (MRI) for one patient (case 1) showed multi-septate cystic lesion, fluid-fluid levels, heterogeneous intermediate to low signal intensity in T1-weighted image (T1WI), and heterogeneous high signal intensity in T2-weighted/short T1 inversion recovery (STIR) with the hemorrhagic component. It shows minimal if any enhancement post-gadolinium administration. The differential was a periosteal-related aneurysmal bone cyst. Multiple enhancing right axillary lymph nodes, the largest measures 1.6x0.9 cm, worrisome for metastasis. MRI was repeated one month later, which shows a significant interval increase in size measuring 3.6x4.5x4.4 cm in anterior-posterior (AP), transverse, and cubic centimeter (CC) dimensions compared to 3.3x2.9x3.1 cm previously (Figure 1). The second patient (case 2) showed oval shape soft tissue mass with lobulated margin, cystic degeneration with multiple areas of fluid-fluid levels suggestive of intra lesion hemorrhagic components, hypo-intense signal intensity in T1, and heterogeneous hyper-intense signal intensity in T2. After administration of IV contrast, enhanced superior tail around the lesion and enhancement of the soft tissue component within the lesion is seen with no enhancement of cystic components differential diagnosis were: nerve sheath tumors such as schwannoma or soft tissue sarcoma such as synovial sarcoma. Multiple enhanced lymph nodes were seen in the left inguinal region, suggestive of metastasis (Figure 2). The third patient (case 3) came with an outside MRI report that was suggestive of synovial sarcoma (Figure 3). All patients showed no bone involvement. No case was initially diagnosed as AFH. CT scan staging showed no metastasis in all three patients, and Triphasic technetium 99 m methyl diphosphonate (MDP) bone scan was done in one patient (case 2) where the tumor showed hyperemia in both flow and blood pool phases (Figure 4). The rest of the study demonstrates physiologic radiotracer distribution for age. 

Needle biopsies confirmed the diagnosis of AFH in all of the three patients. Only one case was initially thought to be synovial sarcoma or spindle cell tumor (case 3). The diagnoses of AFH have reached after fluorescence in situ hybridization (FISH) Interpretation. For the first case, the histopathological reports came as spindle cell/epithelioid neoplasm with cystic changes and hemorrhage in keeping with AFH. For the second case, immunohistochemistry showed tumor cells are positive for desmin, smooth muscle actin (SMA), and focally for S100 protein; negative for epithelial membrane antigen (EMA). Histopathology diagnosis showed mesenchymal neoplasm composed of a mixture of epithelioid and plump spindle cells. Cells show mild nuclear pleomorphism and scattered mitotic activity. There is no definite tumor necrosis identified. Zones of hemorrhage and hemosiderin deposition are noted. Foci of lymphoplasmacytic infiltrate are also noted. Overall histological and immunohistochemical findings are in keeping with AFH. FISH interpretation was positive for EWSR1 (22q12) rearrangement by interphase FISH detected in 63 % of nuclei. Lymph node biopsy: benign & reactive lymphoid tissue with no evidence of malignancy. And finally, for the last case, immunohistochemistry showed tumor cells are positive with anti-EMA and being negative for SMA, S100 protein, AE1/AE3, CD34, and desmin. The biopsy is composed of small fragments of atypical spindle cell neoplasm associated with staghorn-shaped blood vessels. Possibility of synovial sarcoma. FISH Interpretation: negative for SS18(SYT) (18q11.2) rearrangement. Nevertheless, positive for EWSR1(22q12) gene rearrangement in 100 % of nuclei (45 cells). This result, along with the morphologic and immunohistochemical features, supported the diagnosis of AFH. 

All patients underwent surgical excision of the tumor with negative margins, and there were no immediate complications except for one patient who required a skin graft (case 3). Surgical excision specimen subjected to histopathological diagnosis confirming the needle biopsy results. Patients were followed up routinely every three to four months, for the last fifteen (case 1), twenty-six (case 2), and nineteen (case 3) months, with imaging studies to rule out recurrence and metastasis, which were negative. All patients have a full range of motion with no complaints. All cases were summarized in Table 1.

**Table 1:** Summary of cases (n=3). M, Males; F, Females.

Case no.	Sex	Age (year)	location	Size at presentation	Metastasis	intervention	Intraoperative events	Follow-up
1	M	6	Shoulder	3.3 x 2.9 x 3.1	-	Wide surgical excision	non	Uneventful (15 months)
2	F	12	Proximal thigh	5.8 x 4.8 x 5.0	-	Wide surgical excision	non	Uneventful (26 months)
3	F	17	Lateral knee	4.8 x 4.3 x 4.0	-	Wide surgical excision	skin graft	Uneventful (19 months)

## Discussion

Angiomatoid Fibrous Histiocytoma (AFH) accounts for 0.3% of all soft tissue tumors. It is classified by the World Health Organization (WHO) under the category of tumors of uncertain differentiation [7], after being considered a malignant tumor in the past; due to the infrequent malignant behavior [9,10]. AFH is rarely metastasizing with a reported rate of less than 1% and a very low mortality rate [7]. Nevertheless, there is local recurrence in 2-12% of cases [7]. Typically, AFH is presenting as a painless mass in the cutis or subcutis, some patients could present with a paraneoplastic effect like fever, malaise, weight loss, or anemia, and it is usually located in the extremities [10]; this disagrees with what was found in case 2&3, who presented with a painful mass in the medial proximal thigh and lateral knee. It was found the children and young adults are the most common age group to be affected [11]. However, AFH can present in newborns and even elderlies [11].

Diagnosis of AFH is difficult because of the variability of differential diagnosis that includes: aneurysmal bone cysts, schwannoma, soft tissue sarcomas such as synovial sarcoma based on our radiological reports [12]. Other differential diagnoses are hematoma and hemangioma [12]. Radiological modalities in AFH are not specific and would not lead to a specific diagnosis mimicking both aggressive and more benign masses. Colangeli et al. [12] reported that MRI shows cystic areas with an enhancing fibrous pseudo capsule and internal blood-filled foci, as we saw in our cases.

To reach the diagnosis, histopathology is not sufficient by itself. Histopathological features are well summarized as multinodular growth of myoid spindle or histiocytoid cells with a distinctive syncytial appearance, pseudoangiomatous spaces filled with blood and surrounded by tumor cells, a thick fibrous pseudocapsule with prominent hemosiderin deposition, and peritumoral lymphoplasmacytic cuffing with occasional germinal center formation [2]. 

There are no reliable histopathological parameters that predict behavior, and both primary AFH and metastatic AFH are frequently morphologically typical, including the genetically documented case of metastatic AFH [13]. EWSR1 gene rearrangements are associated with a variety of soft tissue neoplasms. This gene can fuse with different partner genes leading to the formation of histologically identical neoplasms. At the same time, it can also fuse with the same genes to cause morphologically and behaviorally different tumors [14].

About half of AFH tumors have desmin expression and sometimes exhibit expression of other markers of myoid differentiation as smooth muscle actin, calponin, and rarely h-caldesmon [15]. In this case series, it was positive for desmin, SMA, S100 protein and negative for EMA in one case, and totally the opposite in the other one, which was positive anti-EMA, and being negative for SMA, S100 protein, AE1/AE3, CD34, and desmin. 

There are three translocations for AFH: t(2:22)(q33:q12) EWSR1-CREB1 fusion gene which is the most common, t(12:22)(q13:q12) EWSR1- Activating transcription factor 1 (ATF1) fusion gene and t(12:16)(q13:p11) FUS-ATF1 fusion gene [1]. In this case, series FISH was positive for EWSR1(22q12) rearrangement. Molecular cytogenetic investigations were found to be vital to confirm the diagnosis of AFH [16]. FISH is used to diagnose EWSR1 rearrangement and reverse transcription-polymerase chain reaction (RT-PCR) to determine the specific fusion transcripts [17]. FISH also was found to have a higher sensitivity for detecting tumors and a better success rate than RT-PCR [18].

In AFH cases, a comprehensive approach by imaging, histopathology, immunohistochemistry, and genetic analysis must be followed to diagnose and differentiate AFH from other soft tissue masses to be properly treated and followed up. Reaching the correct diagnosis of AFH comes with a great value; unfortunately, most presentations are atypical, and as a result, it is frequently misdiagnosed, either for more aggressive tumors like myxoid liposarcoma, metastatic melanoma, Ewing’s sarcoma, myxofibrosarcoma, and synovial sarcoma, or benign tumors like hematoma, hemangioma, lipoma, nerve sheath tumor, and vascular anomalies and other wide differentials depending on the age group [6]. This misinterpretation leads to either over managing and subjecting the patient to unnecessary procedures or under managing the patient and ignoring the risk of recurrence and metastasis, and leading to unfavorable evens.

## Conclusions

The accurate diagnosis of AFH is very important to avoid the risk of metastasis and death from improper treatment. Immunohistochemical studies and molecular confirmation of a positive EWSR1 rearrangement using FISH for AFH are very important for correct diagnosis. This is the first report of three AFH cases in the Kingdom of Saudi Arabia to the best of our knowledge. One was a six-year-old male, and the others were two females of 12 and 17 years.
